# Accuracy of Artificial Intelligence Models in Detecting Peri-Implant Bone Loss: A Systematic Review

**DOI:** 10.3390/diagnostics15060655

**Published:** 2025-03-07

**Authors:** Maryam H. Mugri

**Affiliations:** Department of Maxillofacial Surgery and Diagnostic Sciences, College of Dentistry, Jazan University, Jazan 45142, Saudi Arabia; mmugri@jazanu.edu.sa

**Keywords:** peri-implantitis, periodontitis, periapical radiograph, artificial intelligence, deep learning, convolutional neural network (CNN), machine learning, deep learning, neural networks, dental implant, marginal bone loss

## Abstract

**Background and Objectives:** AI is considered one of the most innovative technologies of this century. Its introduction into healthcare has transformed the industry, significantly impacting various aspects such as education, teaching, diagnosis, treatment planning, and patient care. Researchers have tested the accuracy of various generations of AI models for detecting peri-implant bone loss using radiographic images. While studies have reported promising outcomes, there remains significant potential for improving these models. This systematic review aims to critically analyze the existing published literature on the accuracy of AI models in detecting peri-implant bone loss and to evaluate the current state of knowledge in this area. **Methods:** The guidelines established by the Preferred Reporting Items for Systematic Reviews and Meta-Analyses (PRISMA) were pivotal and provided a framework for preparing, implementing, and recording this systematic review. The protocol for this review was registered in PROSPERO. Four electronic databases (PubMed, Scopus, Web of Science, and Cochrane) were diligently searched on 5–6 January 2025, targeting articles published between January 2000 and December 2024. The PIRD elements (population, index test, reference test, diagnosis of interest) that helped in structuring the protocol of the present review were: P: X-ray images of humans demonstrating the bone loss around the dental implant; I: Artificial intelligence models used for detecting radiographic peri-implant bone loss; R: Expert opinions and reference standards; D: Radiographic peri-implant bone loss. The Quality Assessment and Diagnostic Accuracy Tool (QUADAS-2) was used to assess the quality of each included study. **Results:** Seven studies met the selection criteria and were included in the qualitative analysis. A self-designed table was used to tabulate all the relevant study characteristics. The included studies were reported to have a moderate level of certainty of evidence as assessed by the GRADE assessment. In general, all studies included in this review demonstrated a low risk of bias. Overall accuracy of the AI models varied and ranged between 61% and 94.74%. The precision values ranged from 0.63% to 100%. Whereas sensitivity and specificity values range between 67% and 94.44%, and 87% and 100%, respectively. **Conclusions:** The present systematic review highlights that AI models demonstrate high accuracy in detecting peri-implant bone loss using dento-maxillofacial radiographic images. Thus, AI models can serve as effective tools for the practicing dentist in confirming the diagnosis of peri-implant bone loss, ultimately aiding in accurate treatment planning and improving treatment outcomes.

## 1. Introduction

Advancements in technology have tapped every aspect of human life, including dentistry. New technologies such as CAD/CAM [1,2], 3D printing [3], digital scanners [4], and robots [5] have enhanced traditional dentistry. The adoption of these tools has been shown to improve work efficiency, treatment quality, and overall patient satisfaction [4,5,6,7,8].

For a better understanding of AI, the following aspects should be understood. (A) AI is a machine’s ability that displays self-intelligence, which is acquired through learning from a set of information that can help solve a problem without human intervention [9]. (B) In machine learning, algorithms are used to forecast the results. It is a part of AI. Its purpose is to assist machines in learning from data for resolving problems without human intervention [9,10]. (C) Neural networks in AI work similarly to the human brain by conducting signals. These are a collection of algorithms that process signals through artificial neurons. (D) Deep learning or convolutional neural networks are machine learning components that analyze data in various computational layers for the purpose of identifying patterns to improve object detection features [10,11]. DL automatically gathers traits from different layers and processes complex images, thus helping in object detection. AI involves two main steps: training and testing. Training consists of using a dataset to develop and refine the AI’s specifications, while testing involves using the trained AI to perform designated tasks [9,10].

AI is considered one of the most innovative technologies of this century. Its introduction into healthcare has transformed the industry, significantly impacting various aspects such as education, teaching, diagnosis, treatment planning, and patient care [11,12,13]. The use of AI in dentistry is evolving and is currently used for identification and diagnosis of normal anatomical structures (root morphology [14], tooth identification [15], tooth numbering [16]), pathological conditions (caries [17], peri-apical lesions [18], oral cancer [19], bone loss [20]), for predicting the success of implant-supported restorations [21], in treatment planning [22], in forensic odontology [23], and for educating patients and dentists [24,25]. It is a constantly growing field, which is aiding dentists in managing patients [25].

Dental implants are a common treatment option for rehabilitating some or all of the missing teeth [26,27]. The success of implant-supported restorations lies in various factors, including proper case selection, implant placement, prosthesis fabrication with suitable occlusion and contours, patient medical conditions, and oral hygiene maintenance by the patient [27,28,29].

Peri-implantitis is an inflammatory condition that affects the soft and hard tissues surrounding dental implants, jeopardizing the success of osseointegrated implants [30]. This condition is multifactorial and can be identified through clinical assessments and radiographic examinations of the affected area [30,31]. Radiographic images can reveal bone loss around the dental implant, which is a key indicator of the condition. Marginal bone loss is a vital factor that should be observed constantly [31,32]. Marginal bone loss up to 1.5 mm after initial loading in the first is considered normal [31,32,33,34].

Early detection of these radiographic changes in peri-implant bone areas can help the dentist intervene in a timely manner, preventing bone loss and thus prolonging the success of the implant-supported prosthesis [32,33,34]. The practicing dentist typically evaluates 2D radiographic images to assess 3D bone loss around dental implants. However, there is a risk of errors in interpretation, which may arise from factors such as fatigue, limited knowledge, or a busy schedule. These misinterpretations can lead to incorrect treatment planning [35,36]. The integration of artificial intelligence in the interpretation of radiographic features has been shown to help dentists reduce errors and enhance their diagnostic processes, ultimately lessening their workload [35].

Researchers have tested the accuracy of various generations of AI models for detecting peri-implant bone loss using radiographic images [37,38,39,40,41,42,43]. While studies have reported promising outcomes, there remains significant potential for improving these models. Therefore, this systematic review aims to critically analyze the existing published literature on thEe accuracy of AI models in detecting peri-implant bone loss and to evaluate the current state of knowledge in this area. The null hypothesis framed is that there is no difference in the accuracy of artificial intelligence models in detecting radiographic peri-implant bone loss compared to reference standards.

## 2. Materials and Methods

The guidelines established by the Preferred Reporting Items for Systematic Reviews and Meta-Analyses (PRISMA) [44] were pivotal and provided a framework for preparing, implementing, and recording this systematic review. The protocol for this review was registered in the International Prospective Register of Systematic Reviews (PROSPERO registration number: CRD42025633756). Four electronic databases (PubMed, Scopus, Web of Science, and Cochrane) were diligently searched on the 5th and 6th of January 2025, targeting articles published between January 2000 and December 2024.

### 2.1. Article Selection Criteria

The criteria for inclusion and exclusion are outlined in Table 1.

### 2.2. Exposure and Outcome

In this review, the exposure was the detection or diagnosis of peri-implant bone loss by means of artificial intelligence tools, while the outcome was the detection/diagnostic accuracy of these artificial intelligence tools. The PIRD elements [45] (population, index test, reference test, diagnosis of interest) that helped in structuring the protocol of the present review were, P: X-ray images of humans that demonstrate the bone loss around the dental implant; I: Artificial intelligence models used for detection of radiographic peri-implant bone loss; R: Expert opinions and reference standards; D: Radiographic peri-implant bone loss.

### 2.3. Search Design, Selection of Studies, and Extraction of Pertinent Data

Two reviewers (MHM and MES) independently conducted a systematic search of four selected databases (PubMed, Web of Science, Scopus, and Cochrane) in January 2025 to collect all relevant articles. The PIRD elements (‘Artificial intelligence’, ‘Radiographic Peri-implant bone loss’, ‘Implant’, and ‘detection/diagnostic accuracy’) formed the basis of the search strings, which were used with Boolean operators and truncation for conducting the search. Minor modifications were made in the search strings to meet each database’s requirement. The detailed search strategy is provided in Table 2.

Duplicate titles were removed, and titles and abstracts of the remaining articles were reviewed independently by two reviewers (MHM & AKB). The articles that did not satisfy the selection criteria were excluded. Additionally, the search was extended to gray literature and reference lists of the selected articles manually to augment our search and ensure that no pertinent studies were excluded from inclusion. Subsequently, MHM and MSA independently reviewed the full-texts of the selected articles and identified the eligible articles. Any conflicts between the two reviewers were solved through discussion with the third reviewer (AKB) to reach a mutual consensus. Non-eligible articles were removed, and the reasons for exclusions were recorded. MHM extracted relevant data from the selected studies and organized this information in a tabular form. The extracted information included details related to the authors, year of publication, country where the research was conducted, type and name of algorithm network architecture, architecture depth (number of layers), number of training epochs, learning rate, modality, X-ray collection duration, number of X-rays/areas evaluated, comparator, test group, training/validation number and ratio, accuracy reported, evaluation accuracy/average accuracy/statistical significance, effectiveness of the results, outcomes, authors’ suggestions, and conclusions. A second reviewer (MSA) later verified the charted data.

### 2.4. Quality Assessment of Included Studies

The Quality Assessment and Diagnostic Accuracy Tool (QUADAS-2) [46,47] was used to assess the quality of each included study. The QUADAS-2 tool evaluates the risk of bias and applicability concerns. The risk of bias section has four domains (patient selection, index test, reference standard, and flow and timing). The applicability concern section has three domains (patient selection, index test, and reference standards). A second reviewer (MSA) later verified the charted data.

## 3. Results

### 3.1. Identification and Screening

**Figure 1:**
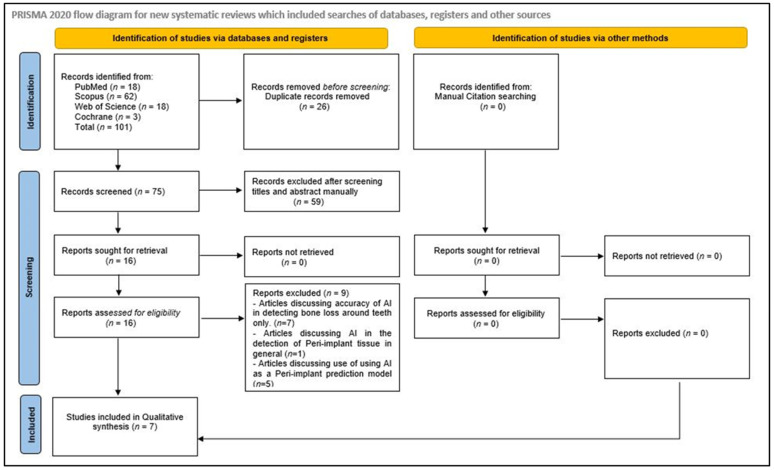
PRISMA 2020 flow diagram depicting the search strategy.

After conducting the initial electronic database search, a total of 101 results were obtained. Twenty-six of these results were identified as duplicates and removed. The remaining 75 articles were screened for eligibility by reviewing their titles and abstracts, resulting in the exclusion of 59 articles that did not meet the inclusion criteria. Sixteen articles were then selected for full-text review. Out of these, seven were rejected because they focused on the application of AI in detecting bone loss around natural teeth rather than around dental implants [20,48,49,50,51,52,53]. Bone loss patterns around natural teeth (periodontitis) and dental implants (peri-implantitis) are different as dental implants lack some of the anatomical structures (like periodontal ligaments, cementum, etc.) present in natural teeth. Additionally, implants, being foreign metallic objects, cannot be directly compared with teeth. Furthermore, one study was excluded because it discussed AI in the detection of peri-implant tissue, in general, without focusing on bone loss specifically [54]. Another study was excluded because it evaluated the accuracy of AI when used as a peri-implant prediction model [55]. Ultimately, seven studies were included in the qualitative analysis [37,38,39,40,41,42,43]. A manual search of references and gray literature did not yield any relevant studies. An excellent agreement (Cohen’s kappa score: 0.88) was found between the two reviewers who performed the full-text review of the sixteen selected studies. Details of the search process are illustrated in a flowchart in Figure 1.

### 3.2. Study Characteristics

**Figure 2:**
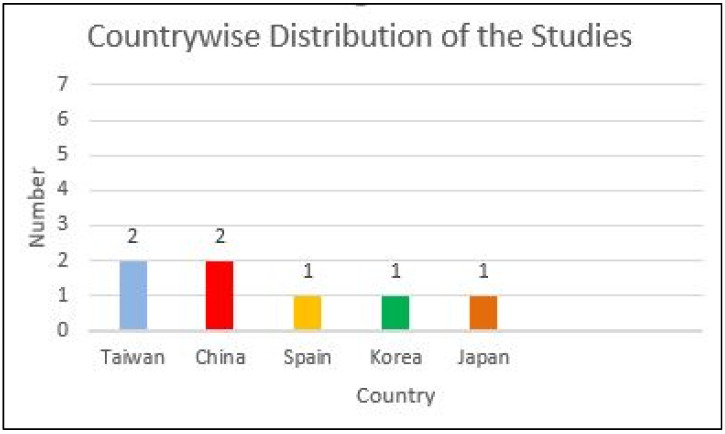
Country-wise distribution of the studies.

Out of the seven selected studies, two were conducted in Taiwan [38,39] and two in China [40,43]. One study each was performed in Spain [37], Korea [41], and Japan [42] (Figure 2). Most of the papers that met the inclusion criteria originated from East Asia (6 out of 7). This occurrence is coincidental, and there is no bias present in the search methodology.

All studies were carried out between 2020 and 2024. Specifically, three studies took place in 2023 [37,38,39], two in 2021 [41,42], and one study each in 2020 [43] and 2022 [40]. Three of the studies [37,38,39,41] utilized two deep learning models: one for detecting implants and another for detecting bone loss. Additionally, one study each used one [40], three [42], and four [43] machine learning models to assess bone loss around dental implants and compare their accuracy in detection. All studies relied on specialist dentists as reference standards, with the number of specialists ranging from one to three. Six out of the seven studies used intraoral periapical radiographs for evaluating bone loss [37,38,39,40,41,42], while one study employed CBCT [43] as the modality for bone loss evaluation using AI. In total, more than 6500 radiographic images were analyzed by AI tools across all seven studies. These images served to train and test the AI models, with the training-to-testing group ratio varying among the studies, ranging from 80:20 [38] to nearly 50:50 [37]. The selected studies reported diagnostic efficiency using various quantifiable outcome measures, including accuracy, precision, sensitivity, specificity, F1 score, AUC, mean error, misdiagnosis rate, positive predictive value, and mean object key point similarity (Table 3).

### 3.3. Assessment of Strength of Evidence

Certainty assessment of evidence from studies included in this review was performed using the Grading of Recommendations Assessment Development and Evaluation (GRADE) approach. Five domains were used to determine the certainty of evidence, which include: Inconsistency, Indirectness, Imprecision, Risk of Bias, and Publication Bias. The levels of certainty of evidence can be very low, low, moderate, and high. For the present systematic review, the included studies were reported to have a moderate level of certainty of evidence as assessed by the GRADE assessment (Table 4).

### 3.4. Accuracy Assessment/Features of the Included Studies

The included studies evaluated the efficiency of detection of bone loss around dental implants using varying outcome measures. Measures used were accuracy [38,39,42], precision [39,42], sensitivity [39,40,43], specificity [39,40,43], F-1 score [39,42], mistake diagnostic rate [40], error [37], omission diagnostic rate [40], positive predictive value [40], mean object key point similarity [41], area under curve [42,43], and recall [42]. The overall accuracy of the AI models varied and ranged between 61% [42] and 94.74% [39]. The precision values ranged from 0.63% [42] to 100% [39]. Whereas sensitivity and specificity values range between 67% [40] to 94.44% [39], and 87% [40] and 100% [39,43], respectively.

### 3.5. Risk of Bias Assessment and Applicability Concern

**Figure 3:**
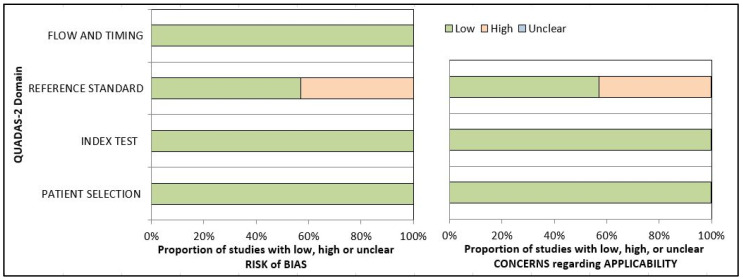
Quality assessment of the individual risk of bias domains and applicability using QUADAS-2 tool.

The QUADAS-2 tool was utilized to evaluate the quality and risk of bias in the studies included in this review [37,38,39,40,41,42,43] (Supplementary Table S1). All the studies used oral–maxillofacial radiographic images of patients as input data for the AI tools, resulting in a low risk of bias (10%) in the patient selection domain. Additionally, all studies followed a standardized protocol for training, leading to a low risk of bias (100%) in the index test domain. However, in three of the selected studies [37,39,42], a single observer performed all the annotations, which resulted in a higher risk of bias (42%) in the reference standard domain. The risk of bias assessment in the flow and timing domain was low (100%) because the procedure for inputting data into the AI model was standardized. The applicability concerns arm showed similar results in terms of risk of bias. Overall, all studies included in this review demonstrated a low risk of bias (Figure 3).

## 4. Discussion

The incorporation of AI in diagnosing various dental conditions by interpreting dental radiographs has helped dentists accurately diagnose pathologies, identify normal and abnormal conditions, and predict the course of the disease [14,15,16,17,18,19,20,21,22,23,24,25]. This advancement saves valuable chairside time for dentists and improves the workflow [56,57].

The use of AI models in detecting and diagnosing peri-implantitis has been reported in multiple studies [37,38,39,40,41,42,43]. Thus, it was necessary to draft a comprehensive systematic review to present an outline of the existing evidence. Our examination focused on how accurately AI models help determine peri-implant bone loss in radiographic images. All the research articles evaluating the accuracy of AI models in detecting/diagnosing peri-implant bone loss were included in this systematic review [37,38,39,40,41,42,43]. In general, this review revealed that using AI to detect and diagnose peri-implant bone loss is an accurate and dependable method. Thus, the tested null hypothesis is rejected. These software can help dentists address the challenges involved in accurate diagnosis and thus can help in precise diagnosis and treatment planning [35,56,57,58,59,60].

Three studies employed two or more AI models to assist in determining peri-implant bone loss [37,38,41]. In general, the first model is used to roughly identify the implant and the implant-supported prosthesis, whereas the second model identifies some significant points that help calculate the loss of bone around the implant. On the other hand, there is only one AI model for bone loss detection in other studies [39]. All the included studies measured the length of the implant not covered by bone and compared it to the implant length covered by the bone to determine the presence of peri-implantitis [37,38,39,40,41,42,43].

Vera et al. [37] used the YOLOv3 AI model for implant localization. This model identifies the implant and prosthesis. Later they used an image understanding-based (IU) AI model, which was used to identify fine lines on implant edges and to identify the intensity of bone changes and the junction between the screw and crown. The AI model computes the distance between these points to calculate bone loss. They reported satisfactory performance of both the YOLOv3 model (0.537 to 0.898) and the IU model (2.63 pixels). Their model quantifies the bone loss around the dental implant in terms of percentage when compared to the entire length of the implant. Chen et al. [38] also combined using YOLOv3 for implant location identification and CNN AlexNet for detecting bone loss. YOLOv3 reported an accuracy of up to 89.31%, whereas AlexNet reported 90.45% accuracy. Their results are the more important evaluation of bone loss up to the first thread of the implant, which is considered a critical indicator of implant stability. Cha et al. [41] used a combination of Residual Learning Neural Network (ResNet) (for identifying the upper and lower jaw) and Mask R-CNN (modified R-CNN architecture) (for bone loss and implant localization). The average precision and recall values of maxillary implant detection were reported to be 0.627 and 0.684, respectively, whereas for mandibular implant detection, these values were reported to be 0.657 and 0.728, respectively. For diagnosing peri-implant bone loss, the mean object key point similarity (OKS) values for CNN for maxillary and mandibular arch were 0.8748 and 0.9029, respectively (total dataset: 0.8885). Meanwhile, for the dentist, the mean OKS values were 0.9012. There were no statistical differences between the OKS values of CNN and the dentist in diagnosing peri-implant bone loss. Lee et al. [39] used only one AI model (YOLOv7) for implant localization and measuring peri-implant bone loss. They reported an accuracy of 94.74% in detecting peri-implant bone loss. Similarly, Liu et al. [40] used one region-based convolutional neural network (R-CNN) (Inception Resnet v2) for implant localization and detecting peri-implant bone loss.

Two articles compared the accuracy of AI-based models in detecting peri-implantitis with dentists [40,41], whereas one each compared the accuracy of three AI models [42] and four AI models [43]. Liu et al. [40] compared R-CNN with two dentists (one resident dentist and the other an MD dentist). Overall, AI reported higher detection rate (sensitivity 67%, specificity: 87%, positive predictor value: 81%, mistake diagnostic rate: 13%) when compared to dentists (sensitivity 93% and 62%, specificity: 64% and 77%, positive predictor value: 69% and 70%, and mistake diagnostic rate: 36% and 23%). Cha et al. [41] reported the mean object key point similarity (OKS) values for CNN to be 0.8885, whereas, for the dentist, the mean OKS values were 0.9012. There were no statistical differences between the OKS values of CNN and the dentist in diagnosing peri-implant bone loss. Mameno et al. [42] compared three CNN models (logistic regression (LR), support vector machines (SVM), and random forests (RF) in predicting and diagnosing peri-implant bone loss. They reported that RF performed the highest in predicting the onset of peri-implantitis (accuracy: 0.70, precision: 0.72), followed by SVM and LR. Zhang et al. [43] compared four CNN models (SVM, artificial neural network (ANN), LR, and RF) for predicting and diagnosing severe peri-implant bone loss. They reported that SVM performed the highest in predicting the onset of peri-implantitis (sensitivity: 91.67%, specificity: 100%), followed by ANN, LR, and RF.

The annotation procedure for training AI models varied in the selected studies. Five out of seven studies used intraoral periapical radiographs (IOPARs) [38,39,40,41,42], one study used both IOPARs and bitewings [37], whereas one study used CBCT [43] for annotation. The sample size also varied among the selected studies, ranging from 2920 [37] to 81 [43]. There was variation in the dentists who performed the selection, image standardization, training, and validation. In three studies, only one specialist dentist was involved in this procedure [37,39,42], whereas in one study each, two specialist dentists [43] and three experienced physicians [38] performed these tasks. In only two studies [40,41], trained maxillofacial radiologists were involved along with other experienced dentist/s in performing these procedures. To homogenize the study protocol, all validations and image selection procedures should involve more than one trained maxillofacial radiologist to minimize the risk of bias. There was variation in training epochs, learning rates, and generations of AI models, adding to the heterogeneity among the selected studies.

The overall accuracy of the AI models varied and ranged between 61% and 94.74%. The precision values ranged from 0.63% to 100%. Whereas sensitivity and specificity values range between 67% and 94.44% and 87% and 100%, respectively. The present systematic review highlights that AI models demonstrate high accuracy in detecting peri-implant bone loss using dento-maxillofacial radiographic images and can effectively assist dentists in diagnosing peri-implant bone loss, which aids in precise treatment planning and enhances treatment outcomes.

### 4.1. Limitations and Strengths

The findings of this systematic review improve our understanding of the role AI models play in detecting peri-implant bone loss. However, it is important to interpret these results cautiously due to several inherent limitations. These include the risk of bias in three out of the seven studies included, the absence of standardized definitions for accuracy assessment parameters, high variability among the articles, the use of different generations of AI models, a limited number of radiographic images, variation in sample size in selected studies, risk of bias in single-annotator studies, variability in image quality, the presence of confounding variables, and concerns regarding generalizability. Other limitations of the review include the inclusion of articles published only in the English language, the search of electronic databases limited to articles published in the last 25 years only (as AI technology might be in the budding phase before this period) and the inability to conduct meta-analysis due to high heterogeneity among the included articles. This review’s key strength includes a systematic and detailed search approach, planned selection criteria, and unbiased article selection protocols. To avoid excluding pertinent articles, the authors reviewed and evaluated all articles related to AI and peri-implant bone loss.

### 4.2. Challenges and Future Directions

With the rapid advancements and growth of AI across all medical fields, it is essential that the studies testing these AI models use standardized protocols to ensure that the outcome of their studies can be generalized effectively. The training of AI models requires a substantial amount of accurate data, highlighting the need for a well-labeled data pool when training these AI models. It is also recommended for studies to utilize validated tools for implementing AI models in real-world settings and to adhere to protocols that ensure the confidentiality of patient data. The studies should have trained oral and maxillofacial radiologists in creating the training datasets and annotation process, thus minimizing the chances of errors being incorporated into the AI models. Studies should provide information on a number of human annotators, their professional qualifications, and steps taken to reduce inter-operator variability. Additionally, most of the current studies rely on two-dimensional intraoral radiographic images for the detection of peri-implant bone loss. AI models should be trained on three-dimensional CBCT images that have greater detail to improve diagnostic accuracy. As the field of AI is evolving rapidly, it is imperative to update these tools to improve the accuracy of diagnosis of the tested conditions.

## 5. Conclusions

In conclusion, the present systematic review covering all the available studies highlights that AI models demonstrate high accuracy in detecting peri-implant bone loss using dento-maxillofacial radiographic images. Thus, AI models can serve as effective tools for the practicing dentist in confirming the diagnosis of peri-implant bone loss, ultimately aiding in accurate treatment planning and improving treatment outcomes.

## Figures and Tables

**Table 1:** Inclusion and exclusion criteria.

Inclusion Criteria	Exclusion Criteria
Studies published in the English language	Studies published in languages other than English
Studies published from January 2000 to December 2024.	Studies published prior to January 2000
Human clinical and in vitro studies	Studies conducted on animals
Studies evaluating the diagnostic accuracy of artificial intelligence models in detecting peri-implant bone loss in human X-ray images.	Studies evaluating the accuracy of AI in detecting bone loss around the tooth rather than around dental implants.
	Studies utilizing AI for implant detection
	Studies employing AI in implant planning and assessing the prognosis of implant therapy
	Studies lacking statistical analysis
	Case reports, chapters in books, editorials, letters to the editor, dissertations, commentaries, opinions, reviews, unpublished studies, incomplete trials, and review articles.

**Table 2:** Search terms and Strategy used for the electronic databases.

Database	Combination of Search Terms and Strategy	Number of Titles
PubMed	(((“peri implantitis” [MeSH Terms] OR “dental implants” [MeSH Terms] OR “bone resorption” [MeSH Terms] OR “marginal bone loss” [All Fields] OR “peri implant bone levels” [All Fields] AND (“artificial intelligence” [MeSH Terms] OR “machine learning” [MeSH Terms] OR “convolutional neural network*” OR “deep learning” OR “Deep Neural Network*” OR “Transfer Learning” OR CNN AND (“Dental X-ray” [All Fields] OR radiography [MeSH Terms] OR “Image processing” [All Fields] OR “smart diagnosis” [All Fields] OR “keypoint detection” [All Fields] OR “Computer vision” [All Fields] OR “computer-aided diagnosis” [All Fields] OR “dental diagnostic imaging” [All Fields] OR “Panoramic image*” [All Fields] OR OPG [All Fields] OR “Periapical images” [All Fields] OR “dental Digital radiograph” [All Fields] AND (Accuracy [All Fields] OR Precision [All Fields] OR sensitivity [All Fields] OR specificity [All Fields] AND ((humans[Filter]) AND (2000/1/1:2024/12/31[pdat]) AND (english[Filter]))) Filters: English, Humans, from 1 January 2000–31 December 2024	18
Cochrane	#1: MeSH descriptor: [Peri-Implantitis] explode all trees; #2: MeSH descriptor: [Dental Implants] explode all trees; #3: MeSH descriptor: [Bone Resorption] explode all tree; #4; (marginal bone loss):ti,ab,kw; #5: (peri implant bone levels):ti,ab,kw; #6: MeSH descriptor: [Artificial Intelligence] explode all trees; #7: MeSH descriptor: [Machine Learning] explode all trees; #8: (convolutional neural network):ti,ab,kw; #9: (deep learning): ti,ab,kw; #10: (Deep Neural Network): ti,ab,kw; #11: (Transfer Learning): ti,ab,kw; #12: (CNN): ti,ab,kw; #13: (Dental X-ray): ti,ab,kw; #14: MeSH descriptor: [Radiography] explode all trees; #15: (image processing):ti,ab,kw; #16: (smart diagnosis): ti,ab,kw; #17: (keypoint detection): ti,ab,kw; #18: (Computer vision): ti,ab,kw; #19: (computer aided diagnosis): ti,ab,kw; #20: (dental diagnostic imaging): ti,ab,kw; #21:(Panoramic image): ti,ab,kw; #22: (OPG): ti,ab,kw; #23: (Periapical images): ti,ab,kw; #24: (dental Digital radiograph): ti,ab,kw; #25: (accuracy): ti,ab,kw; #26: (Precision): ti,ab,kw; #27: (sensitivity): ti,ab,kw; #28: (specificity): ti,ab,kw; #29: #1 OR #2 OR #3 OR #4 OR #5; #30: #6 OR #7 OR #8 OR #9 OR #10 OR #11 OR #12; #31: #13 OR #14 OR #15 OR #16 OR #17 OR #18 OR #19 OR #20 OR #21 OR #22 OR #23 OR #24; #32: #25 OR #26 OR #27 OR #28; #33: #29 AND #30 AND #31 AND #32; [Custom year range: 2000–2024; Language: English]	3
Scopus	(“peri implantitis” OR “dental implants” OR “bone resorption” OR “marginal bone loss” OR “peri implant bone levels”) AND (“artificial intelligence” OR “machine learning” OR “convolutional neural network” OR “deep learning” OR “Deep Neural Network*” OR “Transfer Learning” OR CNN) AND (“Dental X-ray” OR radiography OR “Image processing” OR “smart diagnosis” OR “key point detection” OR “Computer vision” OR “computer-aided diagnosis” OR “dental diagnostic imaging” OR “Panoramic image” OR OPG OR “Periapical images” OR “dental Digital radiograph”) AND (Accuracy OR Precision OR sensitivity OR specificity) AND PUBYEAR > 2000 AND PUBYEAR < 2024 AND (LIMIT-TO (SUBJAREA, “DENT”)) AND (LIMIT-TO (DOCTYPE, “ar”)) AND (LIMIT-TO (LANGUAGE, “English”)) AND (LIMIT-TO (SRCTYPE, “j”))	62
Web of Science (core Collection)	#1 (TS = (“peri implantitis” OR “dental implants” OR “bone resorption” OR “marginal bone loss” OR “peri implant bone levels”)) AND #2 TS = (“artificial intelligence” OR “machine learning” OR “convolutional neural network” OR “deep learning” OR “Deep Neural Network*” OR “Transfer Learning” OR CNN)) AND #3 TS = (“Dental X-ray” OR radiography OR “Image processing” OR “smart diagnosis” OR “keypoint detection” OR “Computer vision” OR “computer-aided diagnosis” OR “dental diagnostic imaging” OR “Panoramic image” OR OPG OR “Periapical images” OR “dental Digital radiograph”)) AND #4 TS = (Accuracy OR Precision OR sensitivity OR specificity) #4 AND #3 AND #2 AND #1 Indexes = SCI-EXPANDED, SSCI, A&HCI, CPCI-S, CPCI-SSH, ESCI, CCR-EXPANDED, Timespan: 2000-01-01 to 2024-07-31 and English (Languages)	18

* Truncation is performed.

**Table 3:** Study characteristics of the included studies.

Author, Year, and Country	Algorithm Network Architecture and Name	Architecture Depth (Number of Layers), Number of Training Epochs, and Learning Rate	Modality	Patient Data Collection/X-Ray Collection Duration	Number of X-Rays/Areas Evaluated (N) Test Group and Training/Validation Number and Ratio	Annotation Performed By	Comparator
Vera et al., 2023, Spain [37]	Two ML models used: Deep learning (DL) object detector (YOLOv3: you only look once): to approximately identify two objects: prosthesis and implant Image understanding-based (IU) process to fine-tune lines on implant edges to identify bone changes (intersections between Implant and crown).	NM	Intraoral radiographs [IOPAR (85%) and bitewing (15%)]	NM	2920 radiographic images (lower jaw) Training: 1460 Test: 1394	Specialist Dentist	EORS
Chen et al., 2023, Taiwan [38]	Two ML models used: CNN YOLOv2: for implant location detection CNN AlexNet: for detecting bone loss	- Depth: NM - Training Epochs: 100 - No. of iterations: 100 - Learning rate: 0.00006	IOPAR	NM	406 radiographic images Training: 80% Testing: 20%	Three physicians with at least 5 years of experience	EORS
Lee et al., 2024, Taiwan [39]	YOLOv7 deep learning network: DL object detector with high speed and accuracy compared to previous versions.	- Depth: - 256 layers - Training Epochs: 1000 - No. of iterations: NM - Learning rate: NM	IOPAR	November 2016 to June 2021	800 peri apical images Training: 600 Validation & Testing: 200	Specialist Dentist	EORS
Liu et al., 2022, China [40]	Inception Resnet v2 (Atrous version) (Region-based convolutional neural networks: R-CNNs): object detector	- Depth: NM - Training Epochs: NM - No. of iterations: 60,000 - Learning rate: 0.0003 to 0.00006	IOPAR	NM	1670 PA images Training: 1370 Validation: 150 Test: 150	One experienced dentist (>5 years of clinical experience and one oral and maxillofacial radiologist)	2 Dentist (Dentist 1: resident dentist, Dentist 2: MD dentist with 2 years of clinical experience, Reference standard: Senior dentist (with more than 5 years of clinical experience)
Cha et al., 2021, Korea [41]	2 ML models used: Residual learning neural network (ResNet): for identifying upper and lower jaw Mask R-CNN (modified R-CNN architecture): implant detection	- Depth: - 152 layers - Training Epochs: NM - No. of iterations: 18,000 - Learning rate: 0.0005 to 0.00005	IOPAR	December 2018 to June 2020	708 PA images (upper: 366; Lower: 342) Training: 508 (upper: 266; Lower: 242) Validation: 100 (upper: 50; Lower: 50) Test: 100 (upper: 50; Lower: 50)	2 Dentist (general practitioner and maxillofacial radiologist)	1 Dentist
Mameno et al., 2021, Japan [42]	Three ML models: LR; SVM RF	NM	IOPAR	November 1996 to December 2012	254 radiographic images Training: 70% Testing: 30%	One Specialist Dentist	EORS
Zhang et al., 2020, China [43]	Four ML models based on the R Programming Language were used: SVM ANN LR RF	NM	CBCT	January 2016 to March 2019	81 radiographic images Training: 70% Testing: 30%	Two Specialist Dentist	EORS
Author, Year, and Country	Evaluation of peri-implant bone loss/resorption		Results (+)effective, (−)non effective (N) neutral	Outcome		Inference/ Author’s suggestions/Conclusions	
Vera et al., 2023, Spain [37]	Error: Mean: 2.63 pixels Standard deviation: 1.28 pixels Average *p* value: 0.0213 (*p* < 0.05 is significant)		(+)effective	As the average *p*-value is less than 0.05, the test is statistically significant. From a clinical point of view: AI is able to accurately detect bone loss due to peri-implantitis.		AI methods can detect bone loss in intraoral radiographs and can assist dental specialists in diagnosing peri-implantitis.	
Chen et al., 2023, Taiwan [38]	Accuracy rate of AlexNet damage detection model: 90.45%		(+)effective	CNN has the ability to determine bone loss around implants with high accuracy.		The CNN model has the potential to improve patient outcomes.	
Lee et al., 2024, Taiwan [39]	Values for recognizing peri-implantitis: Accuracy: Overall: 94.74%; Bone loss: 96.18%; Non-bone loss: 93.42% Precision: Overall: 100%; Bone loss: 100%; Non-bone loss: 100% Sensitivity: Overall: 94.44%; Bone loss: 95.83%; Non-bone loss: 93.06% Specificity: Overall: 100%; Bone loss: 100%; Non-bone loss: 100% F1-Score: Overall: 97.10%; Bone loss: 97.86%; Non-bone loss: 96.43%		(+)effective	CNN model can facilitate the detection of marginal bone loss around dental implant.		AI can help dentists effectively and accurately monitor the condition of patients	
Liu et al., 2022, China [40]	Bone loss around implants: Sensitivity: AI: 67%; Dentist 1: 93%; Dentist 2: 62% Specificity: AI: 87%; Dentist 1: 64%; Dentist 2: 77% Mistake diagnostic rate: AI: 13%; Dentist 1: 36%; Dentist 2: 23% Omission diagnostic rate: AI: 33%; Dentist 1: 7%; Dentist 2: 38% Positive predictive value: AI: 81%; Dentist 1: 69%; Dentist 2: 70% Inter observer agreement (k): AI vs. RS: 0.568 (moderate) Dentist 1 vs. RS: 0.544 (moderate) Dentist 2 vs. RS: 0.383 (fair)		(+)effective	CNN model performance is similar to the resident dentist, but less well than the experienced dentist.		CNN model may facilitate the detection of marginal bone loss around implants.	
Cha et al., 2021, Korea [41]	Mean OKS (object keypoint similarity) CNN: Upper: 0.8748; Lower: 0.9029; Total dataset: 0.8885 Dentist: 0.9012		(+)effective	CNN’s ability to determine the extent of bone loss on IOPA for periimplantitis diagnosis is comparable to dentist		CNN can be used to assist the dentist in diagnosing and categorizing peri-implantitis	
Mameno et al., 2021, Japan [42]	AUC: SVM: 0.64 +_ 0.05; RF: 0.71+_ 0.04; LR: 0.63 +_ 0.05 Accuracy: SVM: 0.63 ^#^; RF: 0.70; LR: 0.62 ^#^ Precision: SVM: 0.64 ^#^; RF: 0.72; LR: 0.63 ^#^ Recall: SVM: 0.62 ^#^; RF: 0.66; LR:0.61 ^#^ f1 score: SVM: 0.618 ^#^; RF: 0.69; LR: 0.612 ^#^		(+)effective	MBL prediction performance: RF > SVM > LR		ML methods have higher accuracy in predicting the onset of peri-implantitis.	
Zhang et al., 2020, China [43]	AUC: SVM: 0.967; ANN: 0.928; LR: 0.906; RF: 0.842 Sensitivity: SVM: 91.67%; ANN: 91.67%; LR: 91.67%; RF: 75% Specificity: SVM: 100%; ANN: 93.33%; LR: 93.33%; RF: 86.67%		(+)effective	MBL prediction performance: SVM > ANN > LR > RF		ML algorithms that utilize the morphological variation in trabecular bone can be used to successfully predict MBL. ML models perform better when compared to the single predictor in predicting the MBL of mandibular implant	

EORS: Expert opinions, reference standards; region-based convolutional neural network: R-CNN; Reference standard dentist: RS; MBL: Marginal bone loss; intraoral Peri Apical Radiograph: IOPAR; Machine Learning: ML; convolutional neural network: CNN; Support vector machine: SVM; Artifcial neural network: ANN; Logistic regression: LR; Random forest: RF; ^#:^ Statistically significant

**Table 4:** Assessment of strength of evidence using GRADE approach.

Outcome	AI Application in Detecting Peri-Implant Bone Loss in Peri-Apical Images [37,38,39,40,41,42]	AI Application in Detecting Peri-Implant Bone Loss in CBCT Images [43]
Inconsistency	Not present	Not present
Indirectness	Not present	Not present
Imprecision	Not present	Not present
Risk of Bias	Present	Not present
Publication Bias	Not present	Not present
Strength of Evidence	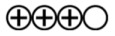	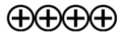

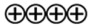
—High evidence 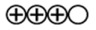
—Moderate evidence.

## Data Availability

The data that support the findings of this study are available from the corresponding authors upon reasonable request.

## Supplementary Materials

The following supporting information can be downloaded at: https://www.mdpi.com/article/10.3390/diagnostics15060655/s1. Supplementary Table S1: Quality Assessment (QUADAS-2) summary of Risk Bias and Applicability concerns.
