# Supplementary material for: Accuracy of Artificial Intelligence Models in Detecting Peri-Implant Bone Loss: A Systematic Review
Source: Diagnostics (Basel). 2025 Mar 7;15(6):655. doi: 10.3390/diagnostics15060655 (PMC11941572; doi:10.3390/diagnostics15060655)
Supplement: Supplementary file 1 [file diagnostics-15-00655-s001.zip › diagnostics-3469126-supplementary.pdf]

Supplementary Table S1. Quality Assessment (QUADAS-2) summary of Risk Bias and Applicability concerns.

Study	RISK OF BIAS				APPLICABILITY CONCERNS		
	PATIENT SELECTION	INDEX TEST	REFERENCE STANDARD	FLOW AND TIMING	PATIENT SELECTION	INDEX TEST	REFERENCE STANDARD
Vera et al., 2023, Spain [37]	☺	☺	☹	☺	☺	☺	☹
Chen et al, 2023, Taiwan [38]	☺	☺	☺	☺	☺	☺	☺
Lee et al, 2023, Taiwan [39]	☺	☺	☹	☺	☺	☺	☹
Liu et al., 2022, China [40]	☺	☺	☺	☺	☺	☺	☺
Cha et al., 2021, Korea [41]	☺	☺	☺	☺	☺	☺	☺
Mameno et al, 2021, Japan [42]	☺	☺	☹	☺	☺	☺	☹
Zhang et al, 2020, China [43]	☺	☺	☺	☺	☺	☺	☺

☺ Low Risk ☹ High Risk ? Unclear Risk
